# Supplementary material for: Acute Effects of Butyrate on Induced Hyperpermeability and Tight Junction Protein Expression in Human Colonic Tissues
Source: Biomolecules. 2020 May 14;10(5):766. doi: 10.3390/biom10050766 (PMC7277647; doi:10.3390/biom10050766)
Supplement: Supplementary file 1 [file biomolecules-10-00766-s001.pdf]

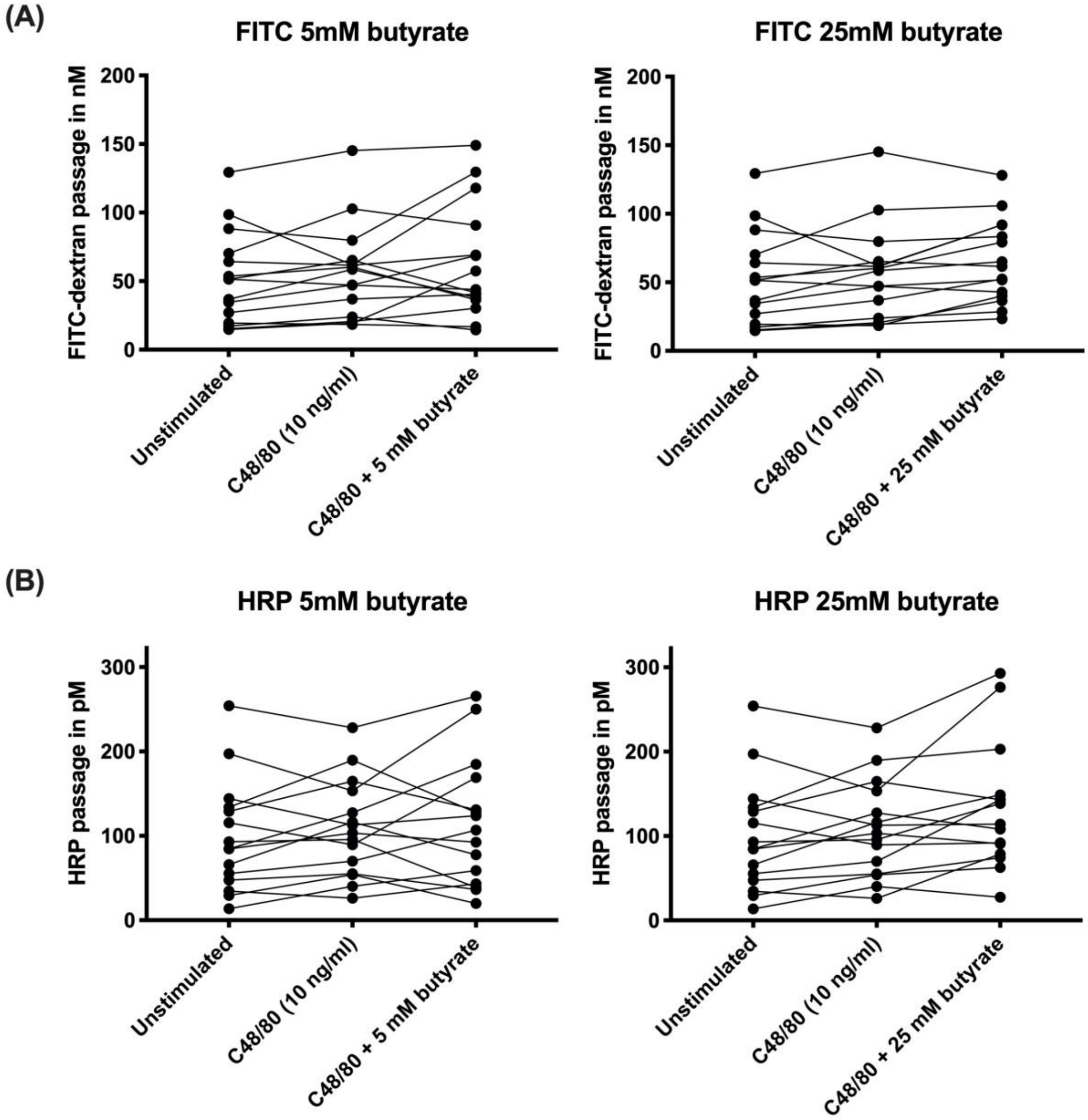

Figure 1. Effects of butyrate on intestinal permeability in colonic biopsies from all participants. Paracellular. permeability (**A**) and transcellular permeability (**B**) are displayed with dots connected by a line for each participant. Biopsies were mounted in Ussing chambers and analyzed in biological triplicates with no stimulation (unstimulated), stimulation with C48/80 (10 ng/ml) alone or in combination with 5 mM sodium butyrate or 25 mM sodium butyrate, respectively. Data from one participant was excluded due to technical problems. $n = 15$

Table 1. Age and sex distribution in the study population and in its subgroups (stressor effect above and below the threshold of 20%).

Participant groups	Age (min–max)	Age (median; IQR)	Sex (female/male)
All participants	24–65	27; 26–33	6/9
Stressor effect ³ 20%	24–39	27.5; 26.25–33.5	4/6
Stressor effect < 20%	25–65	27; 26–31	2/3

Table 2. Absolute transepithelial electrical resistance (TER) values (mean ± s.d.).

Stimulation and treatment	T0	T30	T60
Unstimulated	18.55 ± 4.94	15.43 ± 4.52	14.98 ± 4.40
C48/80	16.27 ± 5.34	14.34 ± 4.80	13.76 ± 4.82
5mM butyrate plus C48/80	16.40 ± 5.79	13.58 ± 5.08	12.99 ± 4.94
25mM butyrate plus C48/80	14.52 ± 5.73	11.34 ± 5.34	10.76 ± 4.87

Table 3. Correlation analysis between age and intestinal permeability and between sex and intestinal permeability.

Tested correlations	Paracellular Permeability (p-value, R2)	Transcellular Permeability (p-value, R2)
Age to permeability (unstimulated)	0.650, 0.016	0.101, 0.194
Age to stressor effect[1]	0.586, 0.023	0.888, 0.002
Age to butyrate effect 5 Mm[2]	0.751, 0.008	0.970, >0.001
Age to butyrate effect 25 mM[3]	0.767, 0.007	0.804, 0.005
Sex to permeability (unstimulated)	0.754	0.770
Sex to stressor effect	>0.999	>0.999
Sex to butyrate effect 5 mM	>0.999	>0.999
Sex to butyrate effect 25 mM	>0.999	>0.999

[1]stressor effect means the ratio of the permeability values of C48/80 vs unstimulated, [2]butyrate effect 5mM means the ratio of permeability values of C48/80 plus 5 mM butyrate vs C48/80, [3]butyrate effect 25mM means the ratio of permeability values of C48/80 plus 25 mM butyrate vs C48/80.
